# Impact of poly-arginine peptides R18D and R18 on alteplase and tenecteplase thrombolysis in vitro, and neuroprotective stability to proteolysis

**DOI:** 10.1007/s11239-022-02642-4

**Published:** 2022-03-19

**Authors:** Bruno P. Meloni, David J. Blacker, Adam B. Edwards, Neville W. Knuckey

**Affiliations:** 1 Perron Institute for Neurological and Translational Sciences, QEII Medical Centre, RR Block, 8 Verdun St, Nedlands, WA 6009 Australia; 2 Department of Neurosurgery, Sir Charles Gairdner Hospital, QEII Medical Centre, Nedlands, WA 6009 Australia; 3 Centre for Neuromuscular and Neurological Disorders, The University of Western Australia, Nedlands, 6009 Australia; 4 Department of Neurology, Sir Charles Gairdner Hospital, QEII Medical Centre, Nedlands, WA 6009 Australia

**Keywords:** R18D, R18, NA-1, Thrombolysis, Alteplase, Tenecteplase, Proteolytic stability

## Abstract

The poly-arginine peptides R18D and R18 represent novel potential neuroprotective treatments for acute ischaemic stroke. Here we examined whether R18D and R18 had any significant effects on the thrombolytic activity of alteplase (tPA) and tenecteplase (TNK) on clots formed from whole blood in an in vitro thrombolysis plate assay. R18D and R18 were examined at concentrations of 0.25, 0.5, 1, 2, 4, 8 and 16 µM during the 1-h thrombolytic assay. We also included the well-characterised neuroprotective NA-1 peptide as a control. R18D, R18 and NA-1 all reduced tPA or TNK percentage clot lysis by 0–9.35%, 0–3.44% and 0–4.8%, respectively. R18D, R18 and NA-1 had a modest and variable effect on the lag time, increasing the time to the commencement of thrombolysis by 0–9.9 min, 0–5.53 min and 0–7.16 min, respectively. Lastly, R18 and NA-1 appeared to increase the maximal activity of the thrombolysis reaction. In addition, the in vitro anti-excitotoxic neuroprotective efficacy of R18D and R18 was not affected by pre-incubation for 1–2 h or overnight with tPA or TNK, whereas only R18D retained high anti-excitotoxic neuroprotective efficacy when pre-incubated in a synthetic trypsin (TrypLE Express). The present in vitro findings suggest that neither R18D or R18 when co-administered with the thrombolytic inducing agents tPA or TNK are likely to have a significant impact when used clinically during clot thrombolysis and confirm the superior proteolytic stability of the R18D peptide.

**Supplementary Information:**

The online version contains supplementary material available at 10.1007/s11239-022-02642-4.

## Highlights

- R18D, R18 and NA-1 do not have a significant impact on tPA or TNK clot lysis in the in vitro assay
- Both R18D and R18 retained neuroprotective properties when pre-incubated with tPA or TNK, but only R18D retained neuroprotective properties when pre-incubated with trypsin
- The results indicate that R18D will retain its neuroprotective properties in vivo when co-administered with tPA or TNK, without interfering with thrombolysis

## Introduction

Globally, stroke is a leading cause of death and disability, with currently no clinically available effective neuroprotective therapeutics. A clinically effective neuroprotective drug has the potential to slow infarct growth and extend the therapeutic time window for alteplase thrombolysis in ischaemic stroke and mechanical thrombectomy for stroke caused by large cerebral vessel occlusion, as well as to further improve the patient outcomes following these endovascular interventions. Despite the availability of endovascular therapies most patients do not receive these interventions due to the delays or inability in accessing medical facilities capable of administering alteplase and or performing thrombectomy, as well as not being suitable candidates to receive these treatments. Therefore, a clinically effective neuroprotective therapeutic also has the potential to improve outcomes in patients who do not receive endovascular reperfusion interventions.

The cationic arginine-rich peptides (CARPs) poly-arginine-18 (R18; 18-mer of L-enantiomer arginine; net charge + 18), its D-enantiomer (R18D) and NA-1 (also known as nerinetide or TAT-NR2B9c; net charge + 7) represent emerging neuroprotective therapies for the acute treatment of ischaemic stroke, especially prior to thrombolysis and endovascular thrombectomy [1]. This group of compounds possess potent neuroprotective properties, with peptide arginine content and peptide positive charge critical for neuroprotection [2–4]. Importantly, CARPs such as R18 possess multimodal cytoprotective mechanisms of action (viz. anti-excitotoxic, anti-inflammatory, antioxidant and toxic molecule scavenging, anti-proteolytic and mitochondrial stabilising; reviewed in depth in Meloni et al. [5] and MacDougall et al. [6]), thus providing the best opportunity for clinical effectiveness. Indeed, subgroup analysis in the ESCAPE-NA-1 trial, revealed that patients undergoing thrombectomy who received NA-1, but not alteplase had significantly better functional outcomes, reduced mortality and reduced infarct volume when compared with patients who received both NA-1 and alteplase [7]. The positive effect of NA-1 in the ESCAPE-NA-1 trial also provides preliminary clinical evidence that a neuroprotective drug can further improve patient outcomes following an endovascular treatment.

Given the potential future application of the R18D, R18 and NA-1 peptides as ischaemic stroke neurotherapeutics, it is important to establish if they interfere with current and future standard of care treatments, namely alteplase (tPA; recombinant tissue plasminogen activator) and tenecteplase (TNK; a modified version of tPA) thrombolysis, which are routinely administered prior to thrombectomy. Similarly, it is also important to determine whether tPA or TNK may cause proteolytic degradation of the peptides directly or through the activation of plasminogen to plasmin, thereby reducing their potential neurotherapeutic efficacy. With respect to the latter, it has already been established that NA-1 and R18, but not R18D are sensitive to proteolytic degradation by plasmin [8, 9], and the reason for the lack of efficacy of NA-1 in ESCAPE-NA-1 participants who received alteplase [7, 8]. In addition, it has been reported that NA-1 does not interfere with the thrombolytic activity of tPA or TNK [10] and that a D-amino acid modified NA-1 (D-Tat-L-2B9c), unlike the unmodified NA-1, retains its neuroprotective efficacy when co-administered with alteplase in a rat embolic stroke model [8].

Therefore, this study had two main aims: (1) examine the effect of R18D and R18 on the thrombolytic inducing activity of tPA and TNK in an in vitro thrombolysis assay, with NA-1 serving as a control; and (2) examine the neuroprotective proteolytic stability of R18 and R18D in an in vitro neuronal excitotoxicity injury model after pre-incubation with tPA, TNK or the protease trypsin.

## Material and methods

### Thrombin, alteplase, tenecteplase and peptides used in the study

Thrombin (Sigma Aldrich, Australia) was resuspended in saline for injection (0.9% sodium chloride; Pfizer, Australia) as an 8 µM stock solution and stored at − 20 °C until use. Recombinant alteplase (10 mg vial; Boehringer Ingelheim, Germany) and tenecteplase (50 mg vial; Boehringer Ingelheim) were resuspended in 10 mL water for injection, aliquoted and stored at − 20 °C until use. The R18 (H-RRRRRRRRRRRRRRRRRR-OH; R = L-arginine) and NA-1 (H-YGRKKRRQRRR-KLSSIESDV-OH) peptides were synthesised by Mimotopes (Australia). R18D (H-rrrrrrrrrrrrrrrrrr-OH; lower case = D-arginine) was synthesised by Auspep (Australia). Peptides were purified by high performance liquid chromatography to 98% purity, and subjected to peptide hydrolysis and amino acid liquid chromatography analysis to obtain a precise measure of peptide content. The peptides were resuspended in saline for injection as 500 µM concentration stock solutions before being stored at − 20 °C until use.

### Blood and plasma collection

Human blood was collected by a qualified phlebotomist and handled using appropriate safety procedures, and the Perron Institute approved the procedure. Blood was obtained from one of the authors (BM) via venepuncture into trisodium citrate (3.2% w/v final) vacuum collection tubes (Greiner Bio One, Austria). Plasma was obtained from whole blood in collection tubes after centrifugation for 5 min at 3500 rpm. Whole blood and plasma were stored at 4 °C before use and used on the day of collection or 1 to 2 days after collection for thrombolysis studies.

### Formation of whole blood halo clot

We utilised a high throughput 96-well plate thrombolysis assay (“halo clot assay”) based on the procedure developed by Bonnard et al. [11]. To reduce assay components the halo thrombus formation procedure was modified by replacing the original thrombotic mixture consisting of recombinant tissue factor and synthetic phospholipids reconstituted in 30 mM calcium, with thrombin, and reducing the pro-thrombotic volume (i.e., thrombin) from 5 to 3 µL and blood volume from 25 to 12 µL. Another modification to simplify clot formation consisted of adding the thrombin (3 µL; 500 nM in saline) to one side of the well and 12 µL of blood to the opposite side of well, before using a heat stretched Pasteur pipette with sealed end to mix the two liquids together using 4 to 5 circular motions around the edge of the well (halo clot). The original methodology involved adding the 5 μL of the clotting mixture to the edge of the well, and then using a micropipette to slowly release the 25 μL of blood to the edge of the well while at the same time maintaining a circular motion. Plates were incubated in a humidified 37 °C incubator for 1 h before use in the thrombolysis assay.

### Halo clot assay and spectrophotometric measurement of thrombolysis

The thrombolysis assay involved adding 30 µL of Tris buffer (50 mM Tris–HCl, 100 mM NaCl, pH 7.4) with or without peptide followed by 55 µL Tris buffer containing tPA or TNK (5 nM final concentration) and 50% human plasma (final plasma concentration during thrombolysis 27.5%). The final concentration of peptide during thrombolysis ranged from 0.25 to 16 µM (note: an intravenous dose of R18D at 350 nmol/kg, which is likely to be neuroprotective [9] achieves a Cmax of 0.7–0.9 µM in healthy male and female rats; unpublished observation). The concentration of tPA and TNK used in assay was based on a dose response study using the halo assay, which resulted in approximately 90% clot lysis after 1-h (Supplementary Fig. 1). Following the addition of tPA or TNK into the wells, clot lysis was monitored over a 1-h period at 37 °C using a plate reader (Cytation5, BioTek, USA) by measuring absorbance at 510 nm every 1 min immediately after orbital shaking (205 rpm for 5 s). Controls consisted of wells with no thrombin (100% clot lysis; L_100%_) and wells with no tPA or TNK (0% clot lysis; L_0%_). At the 1-h time point, the percentage of thrombolysis (%T) in tPA or TNK treated samples (L_TX_) was calculated by transforming absorbance values based on the formula: % T = (L_TX_) − (L_0%_)/(L_100%_) − (L_0%_) × 100. In addition, during the 1-h period of clot lysis lag time and maximal activity (also known as maximum velocity or Vmax) of the thrombolysis reaction were calculated. The lag time is defined as the time interval between the first absorbance reading (time = 0) and the line of maximum slope of the thrombolysis reaction. The maximal activity is calculated from the maximum slope over the time course of the assay and reflects the capacity of an enzyme to catalyse a reaction (i.e., thrombolysis), with higher values representing higher catalytic activity (i.e., tPA/TNK induced plasmin thrombolysis).

For each thrombolysis assay, replicates for control wells (L_0%_ and L_100%_) and tPA or TNK only treated wells consisted of n = 4–8. Replicates for tPA or TNK and peptide treated wells consisted of n = 4. For data analysis and graphical representations of the results, data from three independent thrombolytic assays were pooled. Studies consisted of R18D, R18 and NA-1 examined at the 0.5, 1, 2, 4, 8 or 16 µM concentrations performed concurrently on the same 96-well plate, in respective order. In addition, since R18D is known to be resistant to plasmin degradation [9] and appeared to have the most impact on tPA or TNK thrombolysis, a separate set of three independent assays were performed for this peptide alone at concentrations of 0.25, 0.5, 1, 2, 4, 8 or 16 µM. Furthermore, the second dose study with R18D provided the opportunity to include a lower concentration (0.25 µM) of the peptide and to provide comparative data when only one peptide was examined in the thrombolysis assay per 96-well plate as opposed to examining the three peptides concurrently on the same plate.

### Neuronal cultures

The animal procedures were approved by Animal Ethics Committee of the University of Western Australia (2020/ET000227) and adhered to the Animal Welfare Act 2002 (Western Australia) and the Australian Code for the Care and Use of Animals for Scientific Purposes (8th Ed. 2013). Rat primary cortical cultures were established from embryonic day 18–19 Sprague–Dawley rats (Animal Resources Centre, WA, Australia). Briefly, cortical tissue was dissociated in 2 mL Hanks balanced salt solution (HBBS; Life Technologies, Australia) supplemented with 1.3 mM L-cysteine, 10 U/mL papain (Sigma-Aldrich, Australia) and 50 U/mL DNase (Sigma-Aldrich) by trituration with a fire-polished pipette. Dissociated neurons were transferred to a 15-mL tube containing 13 mL of cold Dulbecco’s modified Eagle’s medium (DMEM; Life Technologies)/10% horse serum (Life Technologies) and centrifuged for 4 min at 300×*g*. Neurons were gently resuspended in 1.5 mL of cold Neurobasal (NB; Life Technologies)/2% B27 supplement (B27; Life Technologies) and the neuronal cell concentration adjusted to 50,000 neurons/70 μL and 70 μL inoculated into each well of a 96-well microtiter plate (Nunc, USA) pre-treated wells. Pre-treated wells were coated with 50 μL of poly-D-lysine (50 μg/mL; 70–150 K; Sigma-Aldrich) for 2–3 h at room temperature before being replaced with 50 μL of NB/B27, 4% fetal bovine serum (Life Technologies), 1% horse serum, 62.5 μM glutamic acid, 25 μM 2-mercaptoethanol, 30 μg/mL penicillin and 50 μg/mL streptomycin and placed in a CO_2_ incubator (5% CO_2_, 95% air balance, 98% humidity, 37 °C) prior to seeding with neurons. Neuronal cultures were maintained in CO_2_ incubator. On day in vitro four, 60 µL fresh NB/B27 containing the mitotic inhibitor cytosine arabinofuranoside (final concentration 0.15 μM; Sigma-Aldrich) was added and neuronal culture were used on day in vitro 11 to 13.

### Exposure of peptides to tPA and TNK

tPA (1000 µg/mL) or TNK (5000 µg/mL) resuspended in water (Baxter) were used to prepare 10 µg/mL (170 nM; note this is 34 times the concentration used in thrombolysis assay) stocks in MEM/2% B27 supplement (MEM/B27). R18 and R18D at a concentration of 2 µM were resuspended in MEM/B27 with or without tPA (170 nM) or TNK (170 nM) and incubated in the CO_2_ incubator for 1 or 2 h or overnight (22–24 h). Following the incubation, 50 µL of the MEM/B27 peptide solution with or without tPA/TNK was used to treat neuronal culture as described below for the glutamic acid excitotoxicity model. MEM/B27 was used for non-peptide treated glutamic acid and non-glutamic acid control neuronal cultures.

### Exposure of peptides to trypsin

As an alternative to trypsin, TrypLE Express (Thermo Fischer Scientific, Australia) a synthetic serine protease with trypsin-like activity was used. TrypLE Express cleaves at the same two amino acid sites (arginine and lysine) as trypsin and has a similar pH activity profile, as well as being more stable at 37 °C [12]. Hereafter, TrypLE Express is referred to as trypsin.

Trypsin solution was added to an equal volume of MEM/2% B27 (T:MEM/B27). R18 and R18D at a concentration of 2 µM was prepared in T:MEM/B27 or HBSS:MEM/B27 (50:50; no trypsin control) and incubated in the CO_2_ incubator for 1 or 2 h or overnight (22–24 h). Following the incubation, 50 µL of the T:MEM/B27 or HBSS:MEM/B27 peptide solution was used to treat neuronal culture as described below for the glutamic acid excitotoxicity model. HBSS:MEM/B27 media was used for non-peptide treated glutamic acid and non-glutamic acid control neuronal cultures.

### Assessment of peptide proteolytic stability in glutamic acid excitotoxicity model

To assess peptide proteolytic stability, R18 and R18D neuroprotective efficacy after incubation with tPA, TNK or trypsin was examined in the glutamic acid excitotoxicity model. Peptides were added to culture wells (96-well plate format) 5 min prior to glutamic acid exposure by removing media and adding 50 µL of media containing R18 or R18D at a 2 µM concentration. To induce excitotoxicity, 50 µL of Minimal Essential Media (Life Technologies)/2% B27 (MEM/B27) containing glutamic acid (200 µM: final concentration 100 µM; L-glutamic acid. L-glutamic acid was prepared as a 10,000 µM stock in water) was added to the culture wells and incubated in the CO_2_ incubator for 5 min (note: peptide concentration reduced by half during this step). After the 5-min exposure, media was replaced with 100 µL of MEM/B27 and cultures incubated for a further 20–24 h in the CO_2_ incubator, before cell viability assessment. Untreated controls with or without glutamic acid treatment underwent the same incubation steps and media additions. NA-1 was not used as a control in the peptide proteolytic stability studies because it displays no to little neuroprotection in the glutamic acid excitotoxicity model [2].

### Neuronal viability assessment

At different times after glutamic acid exposure (e.g., 0.5–4 h and 22–24 h) cultures were examined by light microscopy for qualitative assessment of neuronal cell viability. Based on light microscopy, neuronal viability in the glutamic acid treated culture wells typically ranges from 2 to 10% at 22–24 h. Neuronal viability was quantitatively measured by 3-(4,5-dimethyliazol-2-yl)-5-(3-carboxymethoxy-phenyl)-2-(4-sulfophenyl)-2H-tetrazolium salt (MTS) (i.e., MTS assay, Promega, Australia) absorbance (490 nm) measurements. The MTS assay is a metabolic assay that measures the ability of viable cells to bioreduce the MTS compound into a coloured soluble formazan product that is measured spectrophotometrically. The quantity of formazan product formed is directly proportional to the number of viable cells in culture. In order for MTS absorbance measurements to better reflect neuronal cell viability and based on visual assessment of neuronal cultures after glutamic acid treatment, MTS data were transformed so that the viability of untreated controls is taken as 100% and the viability after glutamic acid treatment is taken as 5%. At least four wells were used in all assays and repeated a minimum of three times independently.

### Statistical analysis

Percentage thrombolysis at the 1-h time point, lag time and maximal activity were analysed by one-way analysis of variance (ANOVA), followed by post-hoc Dunnett’s test to uncover differences between the control (tPA or TNK) and peptide treatment groups (GraphPad Prism; version 9.3.1., GraphPad Software Inc). Neuronal viability data was analysed by one-way analysis of variance, followed by post-hoc Fisher’s PLSD test (StatView; version 4.51, Abacus Concepts Inc). All data are presented as mean ± standard deviation (SD). A value of p < 0.05 was considered significant for all data analysis.

## Results

### Impact of R18, R18D and NA-1 on tPA and TNK clot thrombolysis

#### tPA

**Fig. 1:**
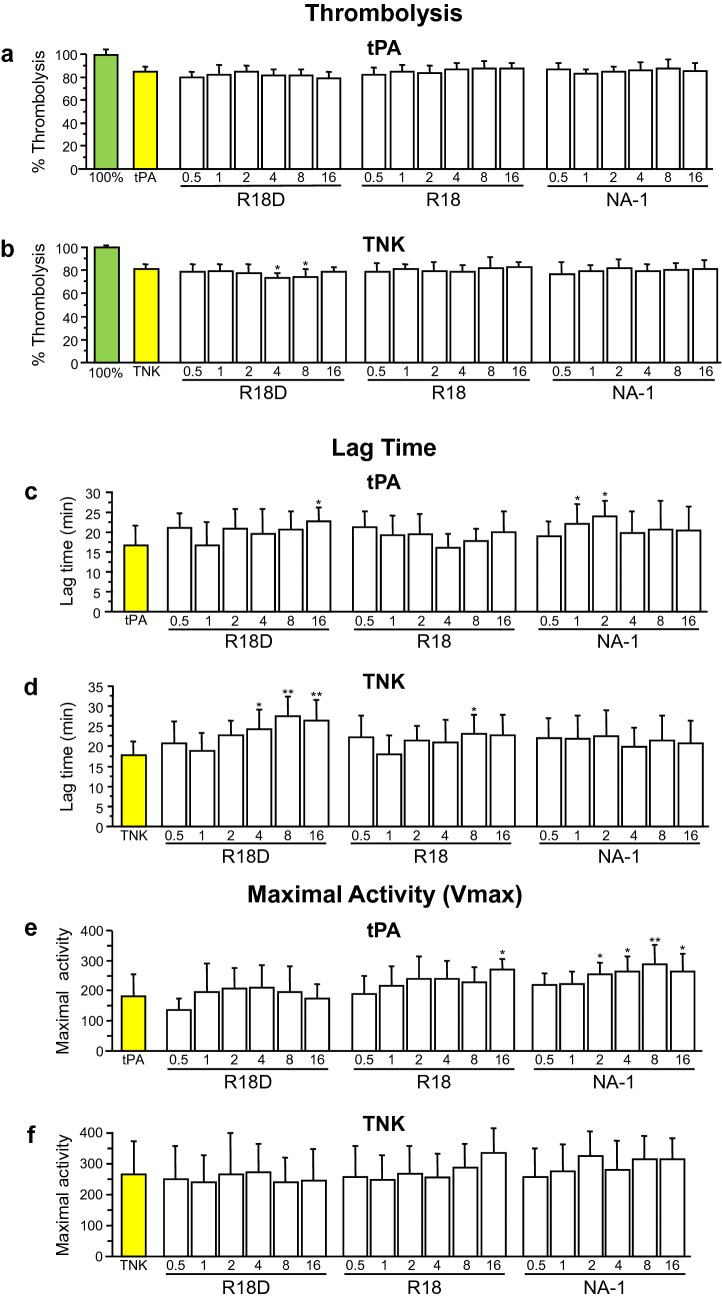
Thrombolysis Halo assay examining the effect of R18D, R18 and NA-1 on tPA and TNK thrombolysis, lag time and maximal enzymatic activity. **a** Percentage tPA thrombolysis. **b** Percentage TNK thrombolysis. **c** Lag time during tPA thrombolysis. **d** Lag time during TNK thrombolysis. **e** Maximal enzymatic activity during tPA thrombolysis. **f** Maximal enzymatic activity during TNK thrombolysis. Data are mean ± SD; N = 8 for 100% thrombolysis (100%), tPA thrombolysis (tPA) and TNK thrombolysis (TNK). N = 4 for all other treatments; *p < 0.05, **p < 0.0001 when compared with corresponding tPA or TNK control

**Fig. 2:**
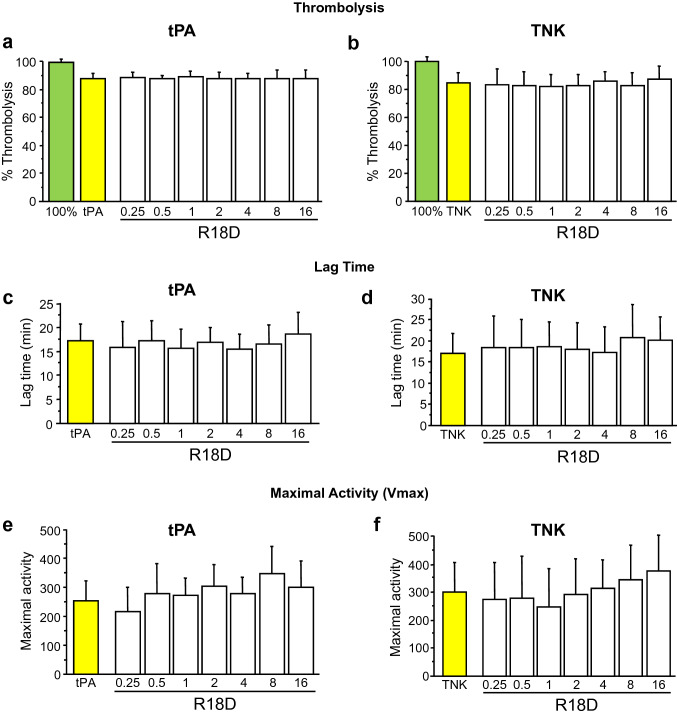
Thrombolysis Halo assay examining the effect of R18D on tPA and TNK thrombolysis. **a** Percentage tPA thrombolysis. **b** Lag time during tPA thrombolysis. **c** Maximal enzymatic activity during tPA thrombolysis. **d** Percentage TNK thrombolysis. **e** Lag time during TNK thrombolysis. **f** Maximal enzymatic activity during TNK thrombolysis. Data are mean ± SD; N = 8 for 100% thrombolysis (100%), tPA thrombolysis (tPA) and TNK thrombolysis (TNK). N = 4 for all other treatments

The overall one-way ANOVA model was statistically significant across the treatments for tPA thrombolysis (F = 2.2, p = 0.004), however subsequent post-hoc analysis showed that R18D, R18 and NA-1 treatments did not significantly inhibit thrombolysis compared with the control (tPA) (Fig. 1a). Significance of the ANOVA model was due to differences between some of the R18D, R18 and NA-1 treatment groups. Similarly, in the separate dose study, R18D did not inhibit tPA thrombolysis (F = 0.65, p = 0.86; Fig. 2a).

#### TNK

The overall one-way ANOVA model was statistically significant across the treatments for TNK thrombolysis (F = 1.66, p = 0.047). R18D at 4 and 8 µM resulted in a modest, but significant decrease in TNK thrombolysis by 9.35% (p = 0.022) and 8.71% (p = 0.044), respectively (Fig. 1b), whereas in the separate dose study, R18D did not inhibit TNK thrombolysis (F = 0.66, p = 0.85; Fig. 2b). Similarly, R18 and NA-1 did not significantly inhibit TNK thrombolysis (Fig. 1b).

### Impact of R18, R18D and NA-1 on tPA and TNK thrombolysis lag time

#### tPA

The overall one-way ANOVA model was statistically significant across the treatments for tPA lag times (F = 2.28, p = 0.003). R18D significantly increased tPA lag time at 16 µM by 5.85 min (p = 0.02) (Fig. 1c). In the separate dose study, R18D did not significantly affect tPA lag time at any concentration (F = 0.47, p = 0.97; Fig. 2c). NA-1 at 1 and 2 µM significantly increased tPA lag time by 5.4 min (p = 0.034) and 7.16 min (p = 0.001), respectively (Fig. 1c), whereas R18 did not significantly increase tPA lag time (Fig. 1c).

#### TNK

The overall one-way ANOVA model was statistically significant across the treatments for TNK lag times (F = 3.43, p < 0.0001). R18D significantly increased TNK lag time at 4, 8 and 16 µM by 6.64 min (p = 0.0037), 9.9 min (p < 0.0001) and 8.76 min (p < 0.0001), respectively (Fig. 1d). In the separate dose study, R18D did not impact TNK lag time at any concentration (F = 0.93, p = 0.54; Fig. 2d). R18 significantly increased TNK lag time at 8 µM by 5.53 min (p = 0.03) (Fig. 1d), whereas NA-1 did not significantly increase TNK lag time (Fig. 1d).

### Impact of R18, R18D and NA-1 on tPA and TNK thrombolysis maximal activity

#### tPA

The overall one-way ANOVA model was statistically significant across the treatments for tPA thrombolysis maximal activity (F = 4.72, p < 0.0001). R18D did not significantly impact tPA thrombolysis maximal activity (Fig. 1e). Similarly, in the separate dose study, R18D did not significantly impact tPA thrombolysis maximal activity at any concentration (F = 0.6, p = 0.9; Fig. 2e). R18 at 16 µM significantly increased tPA thrombolysis maximal activity by 48.36% (p < 0.0001), (Fig. 1e). NA-1 at 2, 4, 8 and 16 µM significantly increased tPA maximal activity by 40.68% (p = 0.014), 45.1% (p = 0.004), 58.65% (p < 0.0001) and 45.65% (p = 0.005), respectively (Fig. 1e).

#### TNK

R18D, R18 and NA-1 did not significantly impact TNK thrombolysis maximal activity (F = 1.21, p = 0.26; Fig. 1f). Similarly, in the separate dose study, R18D did not significantly impact TNK thrombolysis maximal activity at any concentration (F = 0.52, p = 0.95; Fig. 2f).

### R18 and R18D neuroprotective stability after incubation with tPA, TNK or trypsin

**Fig. 3:**
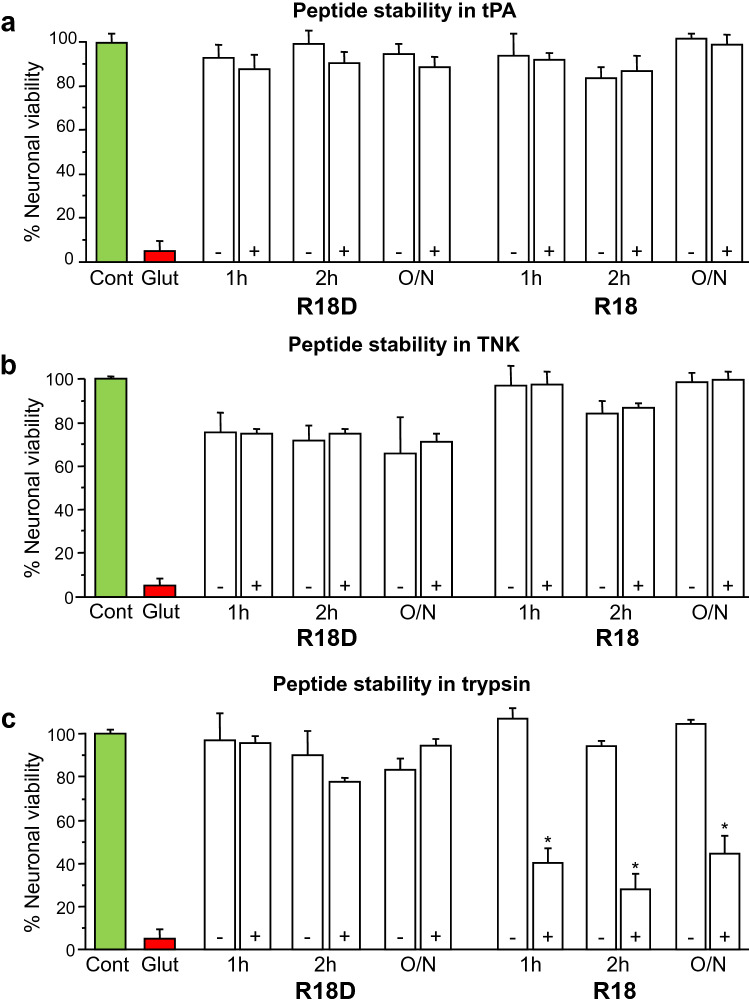
R18 and R18D neuroprotective proteolytic stability after preincubation with tPA, TNK or trypsin. **a** Neuroprotective assessment of peptides in glutamic acid excitotoxicity model after preincubation with tPA (+ : tPA 10 µg/mL) or without tPA ( −). **b** Neuroprotective assessment of peptides in glutamic acid excitotoxicity model after preincubation after preincubation with TNK (+ : TNK 10 µg/mL) or without TNK ( −). **c** Neuroprotective assessment of peptides in glutamic acid excitotoxicity model after preincubation with trypsin (+ : 50% TrypLE Express) or without trypsin ( −) for 1 or 2 h or overnight (O/N: 22–24 h). MTS data were expressed as percentage neuronal viability with control treated (Cont: no glutamic acid exposure) taken as 100% viability and glutamic acid treated (Glut) taken as 5%. Peptide concentration: 2 µM. Values are means ± SE; N = 4–8. *p < 0.05% when compared with neuronal viability in glutamic acid treated neuronal cultures. Note: p < 0.05% also for all other R18 or R18D peptide treatments (± tPA, TNK or trypsin) when compared with glutamic acid treated neuronal cultures

Next, given that peptides may also be degraded directly by tPA and TNK we examined R18D and R18 peptide neuroprotective efficacy after incubation with tPA and TNK. In addition, to further examine R18D and R18 peptide stability the peptides were incubated with trypsin. To assess peptide stability after incubation with tPA, TNK and trypsin, the neuroprotective integrity of R18 and R18D was examined in the glutamic acid excitotoxicity model. R18D and R18 incubated with tPA and TNK for 1 or 2 h or overnight displayed a similar and a high level of neuroprotective efficacy (Fig. 3a, b). In contrast, R18D, but not R18 when incubated with trypsin for 1 or 2 h or overnight displayed a high-level neuroprotective efficacy (Fig. 3c). These findings indicate that R18D and R18 are resistant to degradation by tPA and TNK, but only R18D is resistant to degradation by trypsin.

## Discussion

It is critical that any new acute neuroprotective treatment for ischaemic stroke does not negatively impact the standard of care of tPA thrombolysis or the emerging thrombolytic TNK. In this study, using an in vitro thrombolytic assay, it was demonstrated that R18D, R18 or NA-1 did not have a major impact on clot thrombolysis by tPA or TNK while in the presence of 27.5% plasma. It was however observed that the peptides increased lag time for tPA and TNK thrombolysis, which represents the time interval before active thrombolysis begins. An explanation for the increased lag time induced by R18D, R18 and NA-1 is due to electrostatic interactions between the positively charged peptides and negatively charged fibrin (net charge of − 13.6 per molecule) [13], interfering with the capacity of plasmin to interact with the fibrin clot. It is unlikely the peptides impeded the ability of tPA and TNK to activate plasminogen into plasmin, given that the proteases, like the peptides, are also positively charged (net charge + 5.1) and have a relatively specific peptide cleavage sequence (G, P, Y/broad, K/R↓K/broad, G, G) [14], which would minimise any adverse interactions. Importantly, after thrombolysis commenced, the impact of the peptides on lag time did not appear to have a significant impact on the efficacy of tPA and TNK to lyse clots.

It is important to mention that the results obtained in this study are likely to overestimate any negative effects of R18D, R18 and NA-1 on tPA/TNK lag time and thrombolysis compared with what would occur clinically for several reasons. Firstly, positively charged peptides administered directly into the circulation would have a larger pool of negatively charged plasma proteins such as albumin to bind, and thus reduce any inhibitory electrostatic effects of the peptides on lag time and thrombolysis. In addition, the in vitro thrombolysis assay used in this study was performed in 27.5% plasma whereas whole blood is comprised of 55% plasma. Based on pharmacokinetic studies for NA-1 [7, 8] and R18D (unpublished data), in vivo peak plasma concentration of the peptides and plasma half-life would be in the order of 1–5 µM and 5–15 min, respectively, due to renal, hepatic and splenic clearance, tissue dissemination and enzymatic degradation, compared with the fixed peptide concentration in the closed in vitro system, although R18 and NA-1 are likely to undergo some proteolytic degradation. To this end, the increased resistance of R18D to proteolytic degradation is the likely reason why this peptide at high concentrations demonstrated greater inhibitory effects on lag time. Finally, in the clinical setting early administration of R18D, R18 or NA-1 after stroke onset and prior to the administration of a thrombolytic agent, would reduce the likelihood of peptide-thrombolytic interactions, as well as reduce any negative effects on peptide neuroprotective efficacy. Therefore, based on the information provided above it would be anticipated that the peptides when used clinically at therapeutic doses would have no to little impact of thrombolysis. That said, it is important to mention that while the peptides may not have a significant impact on thrombolysis in vivo, the sensitivity of R18 and NA-1 to proteolytic degradation could impact their neuroprotective efficacy, as observed for the latter peptide in the ESCAPE-NA-1 trial [7].

A potential mechanism that could negatively influence the neuroprotective efficacy of a peptide (and protein) neurotherapeutic when co-administered with a thrombolytic agent in ischaemic stroke is its proteolytic stability. For example, a peptide could be degraded directly by the proteolytic action of tPA and TNK, or indirectly by plasmin following its activation by tPA/TNK. In this study, we have demonstrated that R18D and R18 retain full neuroprotective efficacy in the glutamic acid excitotoxicity injury model after being incubated with relatively high concentrations (170 nM) of tPA and TNK for up to 24 h, indicating that both peptides are resistant to degradation by these enzymes. It has been demonstrated in healthy volunteers that after administration of a 0.5 mg/kg dose of tPA serum concentrations may be as high as 30 nM [15]. This finding is not unexpected because tPA and TNK have a specific peptide cleavage sequence (see above), which is not present on the poly-arginine-18 peptide. Similarly, studies have also demonstrated that NA-1, which does not contain the tPA cleavage site, is not degraded when incubated with the proteolytic enzyme [8]. We also demonstrated that R18D, but not R18 retains high level neuroprotective efficacy in the glutamic acid excitotoxicity injury model when incubated in a proteolytic enzyme with similar properties to trypsin.

The proteolytic stability of R18D when incubated with the trypsin-like enzyme, is in line with previous findings in our laboratory demonstrating that R18D, but not R18, retains its neuroprotective properties in the glutamic acid excitotoxicity injury model when pre-incubated with plasmin [9]. NA-1 is also degraded by plasmin [8]. Together, these findings confirm that peptides synthesised with D-enantiomer amino acids such as R18D, unlike peptides synthesised with L-enantiomer amino acids are highly resistant to proteolytic degradation and are likely to retain their neuroprotective efficacy when co-administered with tPA/TNK thrombolysis in ischaemic stroke.

In some studies, the effects of R18D on thrombolysis differed when the assay was performed with R18D alone and when performed concurrently on the same 96-well plate along with R18 and NA-1. In general, the inhibitory effects of R18D on thrombolysis and lag time were more pronounced when the assay was performed concurrently with R18 and NA-1. One explanation for these differences could be related to the time R18D was exposed to the clot before the addition of tPA or TNK. For consistency, when all three peptides were examined concurrently, the order of addition of the peptides to the wells was R18D, followed by R18, followed by NA-1. Therefore, the few extra minutes clots were exposed to R18D and the rapid nature of electrostatic interaction, may have contributed in part to the modest inhibitor effects for this peptide when examined concurrently with R18 and NA-1.

Another interesting finding was the observation that R18 and NA-1 increased the maximal activity of the tPA thrombolysis reaction. Possible explanations for this effect could be that R18 and NA-1 promote clot destabilisation, stimulate tPA/TNK and or plasmin enzymatic activity or block the activity of plasminogen activator inhibitor 1, with an overall effect of increasing enzymatic clot lysis. Furthermore, given that the proteolysis stable R18D peptide had little impact on maximal activity during clot lysis suggests that R18 and NA-1 peptide proteolytic fragments generated by plasmin are positively influencing tPA/TNK thrombolysis activity. It would therefore be of interest to examine if peptides such as R18 and NA-1 could also influence the enzymatic velocity of other proteolytic enzymes such as calpains and matrix metalloproteinases.

### Limitations of study

This study was performed using in vitro enzymatic and cell culture systems, which provides initial proof of principle data that may translate to the clinical setting. However, while the in vitro thrombolysis system may overestimate any positive or negative effects of the peptides on clot degradation dynamics, it will also be important to confirm the findings of this study in vivo using animals stroke models. For example, studies are planned to examine the neuroprotective efficacy of R18D in a rat ischaemic stroke model when co-administered with tPA. Given that, unlike the L-amino acid NA-1 peptide, a D-amino acid modified version of NA-1 (D-Tat-L-2B9c) with increased proteolytic stability in vitro, also retains neuroprotective properties when co-administered with tPA in a stroke model [8], we anticipate the same will be true for R18D. Finally, data obtained for the in vitro thrombolysis assay was performed using blood samples collected from one individual, and therefore it would be of interest to examine if similar results would have been obtained from blood collected from different individuals.

## Conclusion

In summary, this study has demonstrated that the R18D and R18 peptides are unlikely to have any significant negative effects on tPA/TNK clot thrombolysis and confirm the high proteolytic stability of the R18D isomer. Together, the results indicate that the proteolytic stability of R18D will enable the peptide to retain its neuroprotective properties in vivo when co-administered with tPA or TNK, without interfering with thrombolysis.

## Supplementary Information

Below is the link to the electronic supplementary material.

Thrombolysis Halo assay with tPA and TNK. (**a**) tPA concentration response of percentage thrombolysis measured after 1-hour. (**b**) TNK concentration response of percentage thrombolysis measured after 1-hour. Data are mean ± SD; N = 4. Note: assayed performed without added plasma. Supplementary file1 (PPT 331 kb)
