# Supplementary material for: Impact of poly-arginine peptides R18D and R18 on alteplase and tenecteplase thrombolysis in vitro, and neuroprotective stability to proteolysis
Source: J Thromb Thrombolysis. 2022 Mar 19;54(1):172–82. doi: 10.1007/s11239-022-02642-4 (PMC9259545; doi:10.1007/s11239-022-02642-4)
Supplement: Supplementary file 1 — Thrombolysis Halo assay with tPA and TNK. (a) tPA concentration response of percentage thrombolysis measured after 1-hour. (b) TNK concentration response of percentage thrombolysis measured after 1-hour. Data are mean ± SD; N = 4. Note: assayed performed without added plasma. Supplementary file1 (PPT 331 kb) [file 11239_2022_2642_MOESM1_ESM.ppt]

## Slide 1
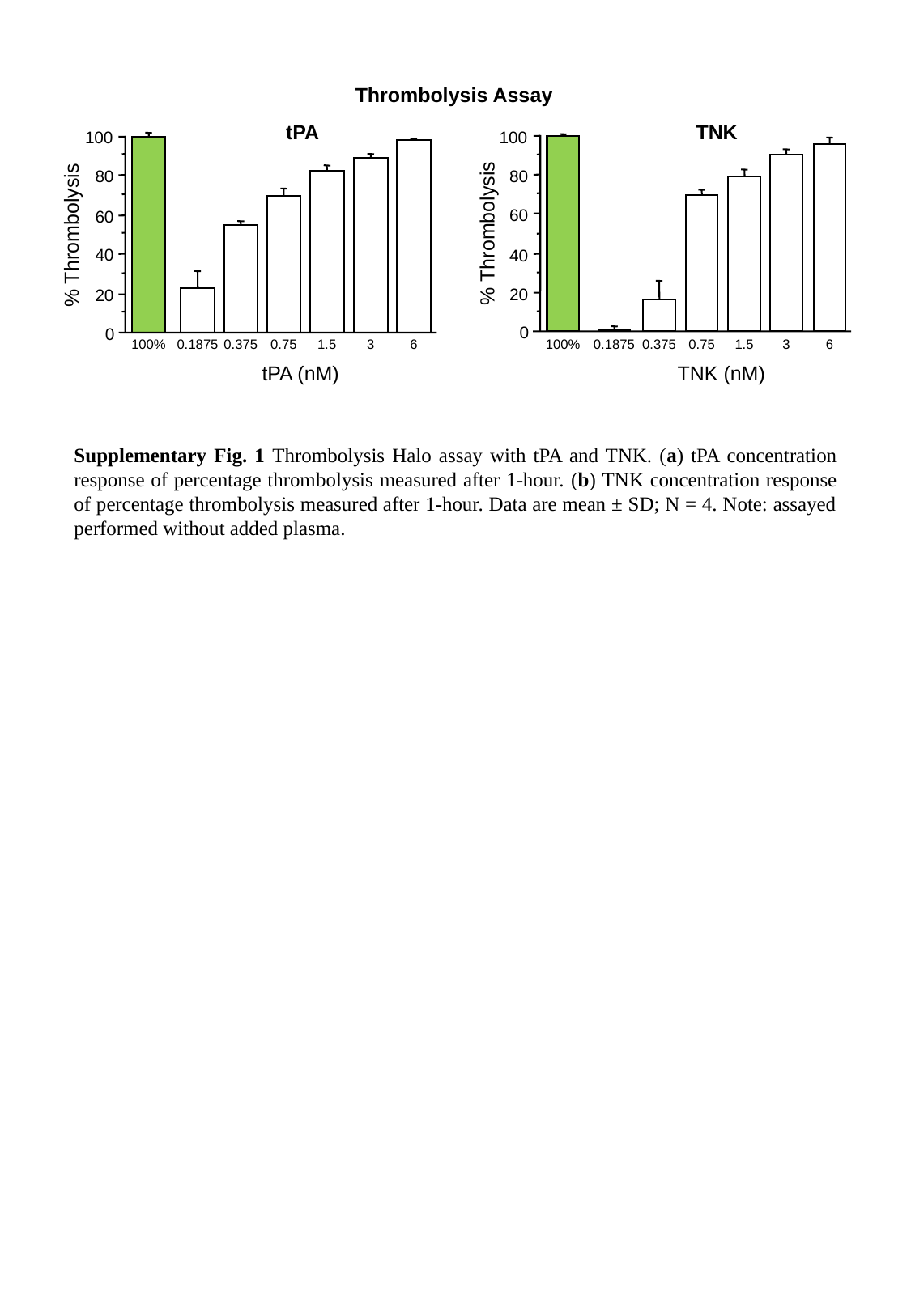

Thrombolysis Assay
tPA
TNK
100
100
80
80
60
60
% Thrombolysis
% Thrombolysis
40
40
20
20
0
0
100%
0.1875
0.375
0.75
1.5
3
6
100%
0.1875
0.375
0.75
1.5
3
6
tPA (nM)
TNK (nM)
Supplementary Fig. 1 Thrombolysis Halo assay with tPA and TNK. (a) tPA concentration response of percentage thrombolysis measured after 1-hour. (b) TNK concentration response of percentage thrombolysis measured after 1-hour. Data are mean ± SD; N = 4. Note: assayed performed without added plasma.
